# Oral health and related outcomes in children and adolescents with cystic fibrosis: a scoping review

**DOI:** 10.1007/s40368-024-00885-8

**Published:** 2024-07-11

**Authors:** D. Chin, L. Ramalingam, J. Harrison, M. Silva

**Affiliations:** 1 Inflammatory Origins, Murdoch Children’s Research Institute, Melbourne, Australia; 2 Melbourne Dental School, University of Melbourne, Melbourne, VIC 3053 Australia; 3 Department of Dentistry, Royal Children’s Hospital, Melbourne, Australia; 4 Department of Respiratory and Sleep Medicine, Royal Children’s Hospital, Melbourne, Australia; 5 Department of Paediatrics, University of Melbourne, Melbourne, Australia; 6 Respiratory Diseases, Murdoch Children’s Research Institute, Melbourne, Australia

**Keywords:** Cystic fibrosis, Oral health, Dental diagnosis, Dental caries, Enamel defects, Gingivitis, Scoping review

## Abstract

**Purpose:**

Good oral health is important for children and adolescents with cystic fibrosis (CF)**. **The purpose of this scoping review is to describe the existing evidence base regarding oral health in children and adolescents with CF and provide recommendations for future research.

**Methods:**

Using a scoping review framework, a comprehensive search was undertaken using medline, embase, and PubMed. The search strategy included broad terms relating to CF, oral health, and children and adolescents and included only papers written in English.

**Results:**

61 articles were included. Topics investigated included dental caries, enamel defects, periodontal health, dental staining, oral health related quality of life, dental management, and dental development of children and adolescents with CF.

**Conclusion:**

Dental outcomes of children and adolescents with CF differ from the healthy population. The current literature describing dental health in children and adolescents with CF includes predominately descriptive analyses. A shift to hypothesis-based studies to explore causal relationships that explain the differences in dental outcomes seen in the CF population offers an opportunity to better understand the problems faced by children and adolescents with CF. Research that actively engages stakeholders, including children and adolescents with CF and their families will enable evidence-based recommendations to improve their oral health.

**Supplementary Information:**

The online version contains supplementary material available at 10.1007/s40368-024-00885-8.

## Introduction

Cystic fibrosis (CF) is a complex, multisystem, life-limiting autosomal recessive disorder (Tümmler 2020; Bell et al. 2020). It is estimated that 162 428 individuals are living with CF worldwide (Guo et al. 2022). Birth prevalence varies between countries and ethnicities and the condition is more common among Caucasians (Walters and Mehta 2007; Yamashiro et al. 1997). It has a wide range of phenotypic presentations but is primarily characterised by progressive respiratory disease, pancreatic exocrine and endocrine failure, and malnutrition (Ahern et al. 2017; Riordan et al. 1989). CF results from mutations in the cystic fibrosis transmembrane conductance regulator (CFTR) gene, affecting the production of the CFTR protein responsible for the transport of chloride and bicarbonate ions across cell membranes (Riordan et al. 1989; Filbrun et al. 2016). Dysfunctional or absent CFTR protein results in hyper-viscous secretions in a number of organs. In the lungs this results in recurrent infections and progressive obstructive lung disease which ultimately leads to respiratory failure- the primary cause of morbidity and mortality (Ahern et al. 2017; Filbrun et al. 2016; Smyth 2005). Advances in molecular therapies have increased the life expectancy of individuals with CF worldwide over the last five decades (Bellis et al. 2007; Hurley et al. 2014; Reid et al. 2011; Smyth et al. 2014; Ruseckaite et al. 2019). A recent therapy, Trikafta, a combination of three oral medications: elexacaftor, tezacaftor, and ivacaftor has contributed to a significant improvement in the management of cystic fibrosis (Zaher et al. 2021). Clinical care for individuals with CF has therefore shifted from focusing on survival, to improving clinical outcomes in all aspects of patients health, and on improving their quality of life (Cohen-Cymberknoh et al. 2011; Pawlaczyk-Kamienska et al. 2019).

The oral health of children with CF involves unique considerations. Although plausible, not all proposed considerations are supported by evidence from primary studies. The frequent consumption of high energy foods and drinks necessary to maintain caloric intake in individuals with CF is thought to increase risk of dental caries (Sutherland et al. 2018; Pitts et al. 2017). Children with CF may also be at increased risk of dental caries because, in general, children with medical conditions are more likely to have developmental defects of enamel which result in weakened tooth structure (Seow 2014). Additionally, frequent use of antibiotics for recurrent respiratory infections, has been proposed to impact oral health including developmental defects of enamel and dental staining (Aps et al. 2002b; Kinirons 1992; Martens et al. 2001; Narang et al. 2003; Peker et al. 2014). Antibiotic use may also alter the composition of the oral microbiome, with potential impacts on susceptibility to dental caries and periodontal disease (Chi et al. 2018). Children and adolescents with cystic fibrosis may also be likely to have behavioural risk factors, relating to diet, oral hygiene and dental service utilisation, especially in later adolescence (Chi et al. 2018).

Advances in the understanding of common oral diseases such as dental caries and periodontology have occurred concurrently with improvements in medical therapy for CF. Given these developments and the broad range of considerations relating to oral health in children and adolescents with cystic fibrosis, it is timely to evaluate the scope of the evidence base and identify gaps in the literature. Unlike a systematic review, which would seek to answer a specific question, this scoping review has been undertaken to describe the breadth of research on the topic, in order to guide future clinical research.

## Purpose

The purpose of this scoping review is to describe the existing evidence base regarding oral health in children and adolescents with CF and provide recommendations for future research.

## Methods

This scoping review utilised the framework developed by Arksey and O’Malley (Arksey and O'Malley 2005). The main research question was: What is the evidence base regarding oral health in children and adolescents with cystic fibrosis? The inclusion criteria were studies (1) involving children or adolescents with CF up to 24 years of age (Sawyer et al. 2018); (2) written in English and (3) relating to CF and oral health. Search terms included dental caries, periodontal health, enamel defects, saliva associated with oral disease, microbiome, antibiotics, oral hygiene, diet, staining, dental development, oral health related quality of life, transition to adult dental services, and/or dental management of children and adolescents with CF.

### Search and selection of sources of evidence

The scoping review protocol was registered on the 20 July 2020 (Chin 2020). There were no limitations on the date of publication, setting, or country of publication. All types of papers (including observational studies, clinical trials, case reports, systematic and narrative reviews, and letters to the editor) written in English were included. Animal studies and studies evaluating saliva for diagnosis and screening of CF that do not include measurement of oral health were excluded.

A comprehensive search was undertaken using Medline, Embase, and PubMed. The search strategy included broad terms relating to CF, oral health, and children and adolescents (Table 1). A search for grey literature was performed by reviewing the reference lists of relevant articles for further publications. The search was initially conducted in 2020 and repeated on 11th August 2022.

**Table 1:** Medline Ovid search strategy

1. exp Tooth abnormalities/
2. exp Tooth Diseases/
3. Oral Health/ or Candidiasis, Oral/
4. exp oral Hygiene
5. exp Periodontal Diseases/
6. (dental or tooth or teeth or caries or enamel or oral-health or oral-hygiene or gingivitis or calculus or plaque or or periodontal or tooth-brush* or toothbrush* or oral-thrush or oral candid*).tw,kf,hw.
7. Cystic Fibrosis/
8. (newborn* or new-born* or baby or babies or neonat* or neo-nat* or infan* or toddler* or pre-schooler* or preschooler* or kinder or kinders or kindergarten* or kinder_aged or boy or boys orgirl or girls or child or children or childhood or pediatric* or paediatric* or adolescen* or youth or youths or teens or teenage or school-age* or schoolage* or school-child* or school-girl* or school-boy* or schoolboy*).af.
9. cystic-fibrosis.tw.kf
10. saliva.tw,kf.
11. *saliva/
12. (1 or 2 or 3 or 4 or 5 or 6 or 10 or 11) and (7 or 9) and 8
13. Limit 12 to English language

Two reviewers (DC and MS) screened the title and abstract of all potentially eligible studies and determined eligibility for inclusion after reviewing the full text.

### Data charting process

Data charting of eligible studies was completed and included (1) bibliographic details including first author, year of publication, country of origin; (2) aims/purpose, population and sample size, methods, outcomes; and (3) key findings that relate to the scoping review questions. Data charting was performed by the primary reviewer. A narrative synthesis of the findings was conducted including year and country of publication, study design, comparison group if applicable, outcome measures and key findings.

## Results

**Fig. 1:**
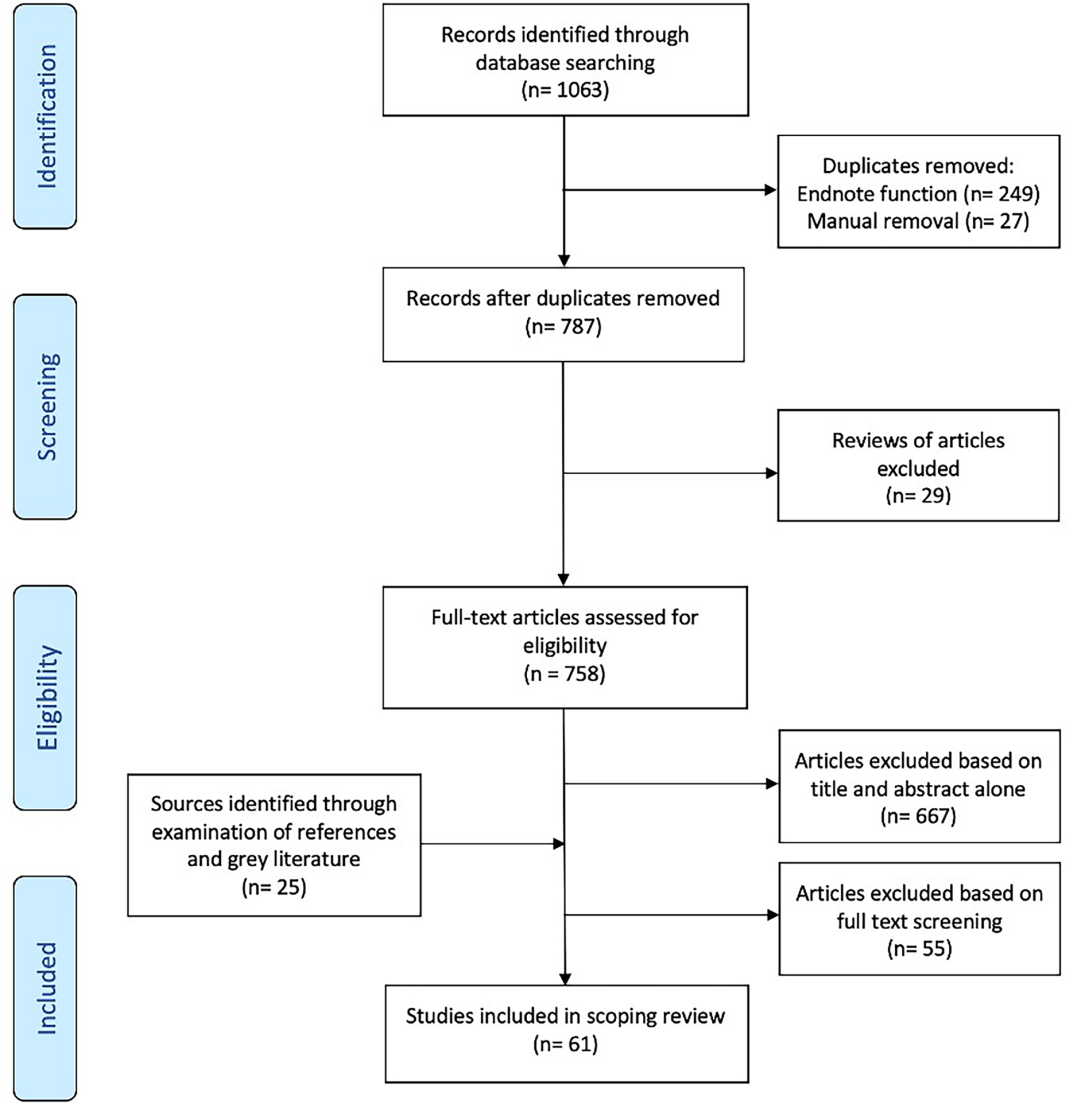
PRISMA flowchart on source selection

Of a total of 1063 potentially eligible peer-reviewed publications identified in the initial search, 61 were included in the final analysis (Fig. 1). One potentially eligible publication was not accessible and was therefore not included (Olejniczak et al. 2003).

### Descriptive analysis

Of the 61 articles included in this scoping review, the majority were cross-sectional observational studies (*n* = 36, 59%). There were also 14 literature reviews, three systematic reviews, two case reports, two descriptive studies, two interventional studies, and two laboratory studies. The majority (*n* = 50, 82%) of the studies were from high-income countries. The earliest article was published in 1962, and the most recent in 2022.

Included studies spanned a range of oral health considerations (Table 2) with dental caries (*n* = 25) and periodontal health (*n* = 19) the most frequently reported. There were no studies on transition to adult dental services. The age of participants ranged from five months to 25 years of age, with one exception of a study with participants aged 0 to 34 years of age (Aps et al. 2001).

**Table 2:** Oral health topics of included studies

References	Country	Study Design	Dental caries in children with CF						Enamel Defects	Perio Health	Dental Stain	Dental Dev	OHRQoL	Transition to Adult Dental Services	Dental Mx
			Dental caries	Micro-biome	Saliva	Anti-biotics	Oral hygiene practices	Dietary habits							
Abu-Zahra et al. (2019)	US	Cross sectional	✓			✓	✓	✓	✓	✓					
Alhaidar (2021)	Saudi Arabia	Case report													✓
Alkhateeb et al. (2017)	US	Cross sectional	✓		✓	✓									
Applebaum et al. (1964)	US	Laboratory study									✓				
Aps et al. (2001)	Belgium	Cross sectional	✓	✓											
Aps et al. (2002b)	Belgium	Cross sectional	✓				✓			✓					
Aps et al. (2002a)	Belgium	Cross sectional	✓							✓					
Atar and Korperich (2010)	US	Literature review													
Attrill and Hobson (1984)	UK	Interventional study													✓
Azevedo et al. (2006)	Brazil	Cross sectional							✓						
Blacharsh (1977)	US	Cross sectional								✓	✓				
Chi (2013)	US	Systematic review	✓												
Chi et al. (2018)	US	Cross sectional	✓	✓	✓	✓	✓	✓							✓
Coffey et al. (2020b)	Ireland	Literature review													
Coffey et al. (2020a)	Ireland	Systematic review								✓					
da Costa et al. (2003)	Brazil	Case report													✓
Dabrowska et al. (2006)	Poland	Cross sectional	✓							✓					
Duruel et al. (2020)	Turkey	Cross sectional								✓					
Fernald et al. (1990)	US	Literature review													✓
Ferrazzano et al. (2009)	Italy	Cross sectional	✓						✓	✓					
Ferrazzano et al. (2012)	Italy	Cross sectional							✓						
Gonçalves et al. (2019)	Brazil	Cross sectional	✓			✓			✓	✓					
Goumghar and Sidqui (2021)	Morocco	Literature review													✓
Harrington et al. (2016)	UK	Literature review													✓
Herman et al. (2017)	Poland	Literature review													
Jagels and Sweeney (1976)	US	Cross sectional	✓			✓			✓	✓	✓				
Kinirons (1983)	UK	Cross sectional	✓		✓										
Kinirons (985)	UK	Cross sectional	✓		✓					✓					
Kinirons (1989)	UK	Cross sectional	✓							✓					
Kinirons (1992)	UK	Cross sectional	✓			✓				✓					
Libman et al. (1979)	US	Descriptive study													✓
Mahaney (1986)	US	Cross sectional										✓			
Martens et al. (2001)	Belgium	Cross sectional	✓							✓					
Moursi et al. (2010)	US	Literature review													✓
Moynihan (2006)	UK	Literature review													✓
Narang et al. (2003)	UK	Cross sectional	✓				✓		✓	✓					
Nazaryan et al. (2019)	Ukraine	Cross sectional								✓					
Nelson et al. (2011)	US	Cross sectional											✓		
Nezon and Liljemark (1980)	US	Cross sectional	✓	✓											
O'Leary et al. (2021)	Ireland	Literature review													✓
Patrick et al. (2016)	US	Cross sectional											✓		
Pawlaczyk-Kamienska et al. (2019)	Poland	Systematic review	✓						✓	✓					
Pawlaczyk-Kamieńska et al. (2022)	Poland	Literature review													✓
Peker et al. (2015)	Turkey	Cross sectional	✓		✓		✓								
Peker et al. (2014)	Turkey	Cross sectional	✓		✓				✓						
Primosch (1980a)	US	Cross sectional										✓			
Primosch (1980b)	US	Cross sectional	✓						✓		✓				
Sarvas et al. (2016)	US	Descriptive study													✓
Sovtic et al. (2019)	Serbia	Literature review													✓
Storhaug (1985)	Norway	Cross sectional	✓				✓	✓	✓						
Storhaug and Holst (1987)	Norway	Cross sectional	✓												
SVSG and Dasaraju (2016)	India	Literature review													✓
Swallow and De Young (1967)	UK	Cross sectional	✓			✓					✓				
Tkachenko et al. (2021)	Ukraine	Interventional study								✓					
Terzian and Schneider (2008)	US	Literature review													✓
Widmer (2010)	Australia	Literature review													✓
Wotman et al. (1973)	US	Cross sectional								✓					
Zegarelli et al. (1963)	US	Laboratory study									✓				
Zegarelli et al. (1964)	US	Cross sectional									✓				
Zegarelli et al. (1965)	US	Cross sectional									✓				
Zegarelli et al. (1962)	US	Cross sectional									✓				

Abbreviations: Dental Dev = dental development; OHRQoL = Oral health related quality of life; Dental Mx = dental management

Many studies (*n* = 31) investigated differences in oral health outcomes between children with and without CF. Eight studies (26%) conducted descriptive, predictive or causal analyses within the CF population and so all participants had CF (Abu-Zahra et al. 2019; Alkhateeb et al. 2017; Gonçalves et al. 2019; Nezon and Liljemark 1980; Patrick et al. 2016; Zegarelli et al. 1964, 1965; Attrill and Hobson 1984). For example, an interventional study investigated the effectiveness of a multidisciplinary CF clinic that offered preventative dental advice and referral to dental services with a comparison group comprised of children with CF who received standard practice (Attrill and Hobson 1984).

Children with CF were recruited from clinical settings such as hospitals in 31 studies, and registries or databases in seven studies. Six studies failed to explain their recruitment protocol. When studies included a comparison group of participants without a diagnosis of CF, they were either siblings (*n* = 4, 13%) (Blacharsh 1977; Jagels and Sweeney 1976; Kinirons 1983, 1985), unrelated children with no known medical conditions (*n* = 21, 68%) (Aps et al. 2001, 2002a, b; Azevedo et al. 2006; Chi et al. 2018; Dabrowska et al. 2006; Duruel et al. 2020; Ferrazzano et al. 2009, 2012; Kinirons 1992, 1989; Mahaney 1986; Martens et al. 2001; Nazaryan et al. 2019; Peker et al. 2015, 2014; Primosch 1980a, b; Wotman et al. 1973; Zegarelli et al. 1962; Tkachenko et al. 2021), or unrelated children and adolescents with another medical condition (*n* = 6, 19%,). Three studies (all by the same research team based in Belgium), had a comparison group of CF carriers who were relatives of the subjects with CF and genetically proven to be heterozygous for mutations of the CFTR gene (Aps et al. 2001, 2002a, b).

## What is the evidence base regarding oral health in children and adolescents with cystic fibrosis?

### Dental caries

Two systematic reviews were included (Chi 2013; Pawlaczyk-Kamienska et al. 2019) and both suggest the prevalence of dental caries in the CF population is lower or similar to unaffected children. However, within the CF population the prevalence of dental caries in the permanent dentition seems to be higher compared to the primary dentition.

The most recent systematic review from 2019 included nine publications with a total of 439 children with CF and 467 unaffected children (Pawlaczyk-Kamienska et al. 2019). These nine publications reported the prevalence of dental caries in the CF population was either lower than or similar to a comparison group (Pawlaczyk-Kamienska et al. 2019). The earlier systematic review conducted in 2013 included 15 studies, seven of which examined the prevalence of dental caries in subgroups by age or dentition (Chi 2013). Six of these studies reported a possible age-related difference in caries risk between children and adolescents with CF. The authors proposed that dental caries risk increases with age due to changes in prescribed CF medications. In childhood, children with CF are more likely to receive beta-lactam penicillin antibiotics targeting *Staphylococcus aureus* which also target bacterial species involved in the pathogenesis of dental caries. However, the authors propose that these protective influences are reduced by adolescence, when an increased prevalence of *Pseudomonas aeruginosa* in the sputum of children and adolescents with CF results in an increased use of aminoglycoside antibiotics (Chi 2013).

Dental caries is a multi-factorial disease, and factors such as the oral microbiome, saliva composition, sugar consumption, antibiotic use, and oral hygiene practices may influence dental caries in children and adolescents with CF.

A total of three studies were identified that investigated the role of the oral microbiome in influencing caries risk of children with CF, compared to children without CF (Aps et al. 2001; Chi et al. 2018; Nezon and Liljemark 1980). Using bacterial culture of *Streptococcus mutans* from saliva or plaque, all three studies (participants aged 6–34 years) failed to demonstrate evidence that differences in *S. mutans* levels explained any differences in dental caries rates (Aps et al. 2001; Chi et al. 2018; Nezon and Liljemark 1980). There were no comprehensive microbiome studies conducted to date, and no studies used deoxyribonucleic acid based studies (16S ribosomal ribonucleic acid or metagenomics).

Associations between the composition of saliva and dental caries rates were explored by six studies (Alkhateeb et al. 2017; Chi et al. 2018; Kinirons 1983, 1985; Peker et al. 2015, 2014). Five of these studies demonstrated differences in saliva flow, buffering capacity, pH, ion composition, and antimicrobial peptide levels in children with CF compared to unaffected children (Chi et al. 2018; Kinirons 1983, 1985; Peker et al. 2015, 2014). Two studies found children with CF had significantly higher salivary pH and buffering capacities in stimulated whole saliva compared to the non-CF children (Kinirons 1983, 1985). They noted lower caries rates (decayed missing filled–dmf/DMF) in children with CF, with greater differences in younger children aged 1–5 years (0.50 ± 0.25 (standard error—SE) for CF vs 1.77 ± 0.50 (SE) for non-CF) which decreased as they got older: 6–10 years (1.54 ± 0.19 (SE) for CF vs 2.04 ± 0.31 (SE) for non-CF) and 11–15 years (3.07 ± 0.50 (SE) for CF vs 5.38 ± 0.70 (SE) for non-CF). In the 1–5 and 11–15 year age groups, the differences in caries rates reached statistical significance (*P* < 0.05), with a trend in the same direction in the 6–10 year age group. The authors proposed an association between alterations in salivary properties (increased salivary pH and buffering capacities) and low caries rates, however no statistical analysis was undertaken to examine this association (Kinirons 1983, 1985).

Two studies (Kinirons. 1992; Chi et al. 2018) attempted to evaluate whether different patterns of antibiotic use could explain the differences in dental caries outcomes in children with CF. An inverse relationship between cumulative antibiotic use (the percentage of months on antibiotic therapy since six years of age) and dental caries prevalence was reported in children aged 8–18 years with CF (Kinirons 1992). There are no longitudinal studies of children with CF and antibiotic use that includes measurements of dental caries at multiple time points to explore age-related changes in risk.

A single study was identified that evaluated the association between oral hygiene practices and dental caries in children and adolescents with CF (Aps et al. 2002b). This study examined the effects of oral hygiene habits on caries rates between CF homozygotes, CF heterozygotes, and non-CF children, and found very few differences in their oral hygiene habits to explain the lower caries prevalence in the CF homozygotes. Data relating to oral hygiene practices were collected in two other studies (Chi et al. 2018; Storhaug 1985). Only one of these reported their findings and did not find associations between tooth brushing time and tooth brushing frequency with dental caries incidence (Chi et al. 2018).

The role of dietary habits in dental caries in children with CF were explored in one study (Chi et al. 2018). Dietary habits were recorded as part of a parental/patient questionnaire which collected details of the frequency of sweet drinks/foods. The study found consuming non-diet carbonated drinks and sugar sweetened beverages more than four times per week, and consuming fewer than 5–7 serves of vegetables per week, was positively associated with dental caries prevalence within the CF population (Chi et al. 2018).

### Enamel defects

A 2019 systematic review of six publications that included a total of 208 participants with CF examined enamel defects including hypomineralisation and hypoplasia and used a variety of indicators of these defects including modified-DDE (Developmental Defects of Enamel), the European Academy of Paediatric Dentistry molar incisor hypomineralisation (MIH) criteria, or limited other study-derived methods (Pawlaczyk-Kamienska et al. 2019). Five studies in the review reported a higher prevalence of enamel defects in the permanent dentition of children and adolescents with CF compared to children with no known medical conditions (Ferrazzano et al. 2009, 2012; Azevedo et al. 2006; Narang et al. 2003), including one which did not carry out any statistical analysis (Dabrowska et al. 2006). The one study which did not report a higher prevalence of enamel defects had a limitation of low participant numbers. Whilst the prevalence of DDE was similar between the two groups, MIH was noted in 20% of children with CF (*n* = 6) with five of the six children having mild defects; one of the six children with moderate defects, and none with severe defects. In comparison, MIH was noted in 23% of children without CF (*n* = 7) with all having only mild defects and no moderate or severe defects (Peker et al. 2014). No differences were found in the primary dentition between the healthy and comparison groups based on the evidence from three studies.

The aetiology of enamel defects in children with CF was explored by one study where 50% (*n* = 10) of the CF cohort had enamel defects compared to 10% of a general population, and a positive association was found between number of antibiotic courses with frequency and severity of enamel defects (Abu-Zahra et al. 2019).

### Periodontal health

A systematic review in 2020 which included 13 studies and 792 individuals with CF found CF participants had lower or similar levels of plaque and gingivitis, and higher levels of dental calculus, compared to a comparison group (Coffey et al. 2020a). A systematic review in 2019, including six studies with a total of 439 children with and 467 children without CF, did not find evidence that a diagnosis of CF leads to increased levels of plaque, calculus or gingival bleeding in those under 18 years of age (Pawlaczyk-Kamienska et al. 2019). However, those older than 18 years demonstrated a significantly lower level of gingival bleeding in the CF group. Both systematic reviews conclude there is insufficient available data to provide an objective assessment of the relationship between CF and periodontal disease due to the lack of specialized tests, including microbial analyses, utilised in studies to date.

One study sought to explain the effect of CF on periodontal outcomes. The authors reported an inverse relationship between the frequency of antibiotic use in the preceding month and levels of plaque and gingivitis (Kinirons 1992). Lower gingival and plaque indexes were found in children and adolescents with CF compared to matched healthy controls with the greatest difference found in those with the highest use of antibiotics in the preceding month (Kinirons 1992).

### Dental staining

Nine studies, spanning from 1962 to 1980, described dental staining in children and adolescents with CF. These studies included 393 individuals with CF and found that most children and adolescents with CF have some degree of dental staining, often associated with a positive history of tetracycline administration. Two studies investigated the relationship between dosage, duration or type of tetracycline used and resultant tooth discolouration (Swallow and De Young 1967; Zegarelli et al. 1963). An association between tetracycline use and dental staining was found with 37–54% of children with CF having discoloured teeth, however there was no consistent relationship found between dosage, duration or type of tetracycline and dental staining.

### Dental development

Two US cross sectional studies reported a delay in dental development of between 2 and 10 months in children and adolescents with CF by comparing their radiographically determined dental age to their chronological age (Mahaney 1986; Primosch 1980a). No correlation was found between delays in dental development and pulmonary disease severity or age at CF diagnosis (Mahaney 1986).

## Oral health related quality of life

Two articles, both from the United States, examined oral health related quality of life in children and adolescents with CF as part of a wider study of children with special health care needs (Nelson et al. 2011; Patrick et al. 2016). They found children with CF had few unmet dental needs and encountered fewer barriers to care compared to other children with significant special health care needs (e.g. Children with cerebral palsy, autism, developmental delay, and Down syndrome (Nelson et al. 2011). However the study did not include children without medical needs. Patrick et al. found Caucasians with CF reported better oral health than other races within the US including African-American, Latino and Asian. They also noted those who took a greater number of medications reported a better oral health related quality of life than those on fewer medications, and adolescents with CF reported poorer oral health related quality of life than their younger counterparts in the domains of social-emotional well-being and self-image (Patrick et al. 2016).

## How should children and adolescents with cystic fibrosis be dentally managed?

There were no publicly available guidelines or protocols identified, however some commentators have provided recommendations to guide the general dental management of patients with CF (Alhaidar 2021; Goumghar and Sidqui 2021; Harrington et al. 2016; O'Leary et al. 2021; Pawlaczyk-Kamieńska et al. 2022; Svsg and Dasaraju 2016; Terzian and Schneider 2008; Widmer 2010). Generic information about how patients with CF should be managed were listed in narrative reviews, but most were not exclusive for patients with CF. Some specific advice for aspects of dental management in patients with CF including sedation risk, general anaesthetic precautions, and dental appointment precautions were provided in one article (Goumghar and Sidqui 2021). Regular dental checks and preventative counselling, including age-specific counselling on risk factors such as malocclusion, pit and fissure sealants and dry mouth was recommended in another article, (Pawlaczyk-Kamieńska et al. 2022).

A UK-based interventional study of 30 children with CF found that inclusion of preventative dental advice and referrals to dental services provided by dental professionals in a multidisciplinary clinic resulted in improved dental outcomes. Reductions in plaque and gingivitis levels, as well as an increase in the number of restored teeth was achieved through visits to a dentist and implementation of oral hygiene advice (Attrill and Hobson 1984).

## Discussion

**Fig. 2:**
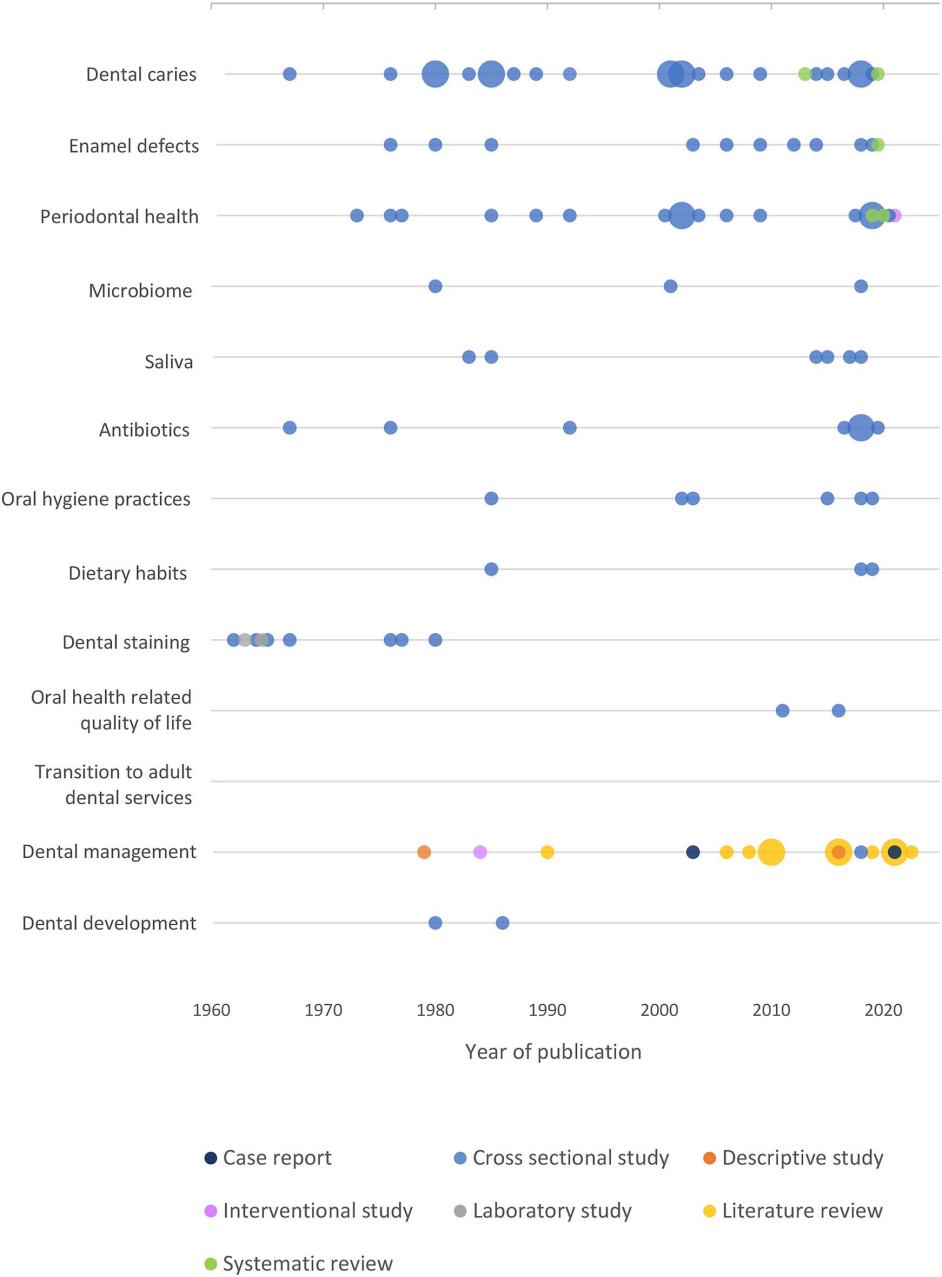
Bubble plot—Publications by topic, study design and year

This scoping review describes the evidence relating to the oral health of children and adolescents with cystic fibrosis (cf. Fig. 2). Much of the evidence was seeking to understand if children and adolescents with CF have higher levels of oral disease than those who do not. These studies were descriptive by nature of the study design and seeking to understand the risk factors and behavioural and biological influences that may be driving differences in oral health between children and adolescents with CF and those without. There was considerable variation in the methodology and although the present study was not a systematic review, such heterogeneity may make it difficult to synthesise results, for example in systematic reviews. There was little interventional research and few qualitative studies that undertook in-depth investigation of the drivers of these potential differences or sought to address the needs of children and adolescence with CF and their families. There is opportunity to explore knowledge gaps in this area particularly relating to transition to adult dental services and management of children and adolescents with CF. No formal guidelines or protocols exist for the dental management of children and adolescents with cystic fibrosis. Recommendations for management of children with cystic fibrosis include an increased focus on prevention, and multi-disciplinary care.

A strength of our study is inclusion of the age of adolescence up to and including 24 years. This is reflective of the changing nature of adolescence to be reflective of the growth and identifications of this stage in life (Sawyer et al. 2018). This extended definition of adolescence is particularly relevant to CF, as evidence is continuing to emerge of an age-related heterogeneity in oral health. This scoping review adopted a transparent and robust methodology and provides a substantial appraisal of the evidence base. In mapping the evidence we have identified potential questions that may be suitable for systematic reviews in the future, but there is a current need for more original research before this can be done. For example, given the critical role of the microbiome and saliva in oral diseases such as dental caries, future research may be beneficial in understanding and improving the dental management of this children and adolescents with CF.

The limitations of this study must be acknowledged. The breadth of included topics is wide, and it is therefore not intended to answer specific questions relating to the oral health of children and adolescents with CF. To keep the project feasible, we only included certain topics related to oral health. We acknowledge that by limiting the scope of the review, some potentially relevant information may not have been captured, for example sedation and GA. Our search of the literature was also focussed only on electronically available literature in English language, therefore articles which were not in this format may have been missed. The search was however comprehensive and is therefore a representative map of the available literature.

By reviewing the existing evidence base we have identified some areas where there is ample research and other areas where questions remain unanswered and further research is required. This review confirms there is steady interest and research for oral health and CF, particularly in descriptive studies. Although such descriptive studies can offer valuable insights into the population of interest, findings are not usually translatable nor are they likely to lead to changes in practice or policy. Undertaking such impactful research is time consuming, requires a range of skills and expertise and expensive. Additionally, in order to be truly impactful, research requires genuine engagement with consumers. Future formulation of a guideline for CF dental care with input from the multidisciplinary teams may prove beneficial (National Health and Medical Research Council 2019). Such resources will enable better oral health engagement of health professionals with children and adolescents with CF and families, develop greater interactions with dental professionals and therefore improve the care and dental outcomes of these patients.

There are many descriptive studies that evaluate whether certain oral conditions are more common in children and adolescents with CF, and any future such studies should consider the limitations described in this scoping review. Longitudinal studies which are well-designed to identify critical time points, behaviours, or protective factors which are related to having CF that may have clinical implications on the management of these individuals would be beneficial. Qualitative research may provide valuable insights into the problems from a patient perspective and how they and their families feel about their dental care, an existing gap in the literature. More health services research investigating the delivery of care to this population and their families would be beneficial. It is important to acknowledge the multiple influences on the oral health of children and adolescents with CF. The evidence seems to group CF into a single entity however future research may be more impactful if it considers risk as dynamic matter that acknowledges the multiple influences on oral health which can alter at different points. For instance, considering the intersection of having CF and other factors such as age and being in adolescence, socio-economic status, mental health and how these risk factors may vary over time for an individual. It may be challenging to undertake this type of research given the relatively small population group. The use of patient registries or digital health may offer opportunities for multi-site, international studies. Additionally, it may help eliminate some of the sources of biases that exist in current CF studies which recruit predominately from high-income countries and fail to capture data from low to middle income countries.

## Conclusion

The evidence relating to the oral health of children and adolescents with cystic fibrosis is mostly descriptive, reporting on differences in behavioural and biological factors between children and adolescents with CF and those without. Future research that focues on topics such as as transition to adult dental services and management of children and adolescents with CF may address knowledge gaps, support development of guidelines and improve oral health of children and adolescents with CF.

### Supplementary Information

Below is the link to the electronic supplementary material.

Supplementary file1 (XLSX 63 KB)

## Data Availability

The authors confirm that the data supporting the findings of this study are available within the article and its supplementary materials.
